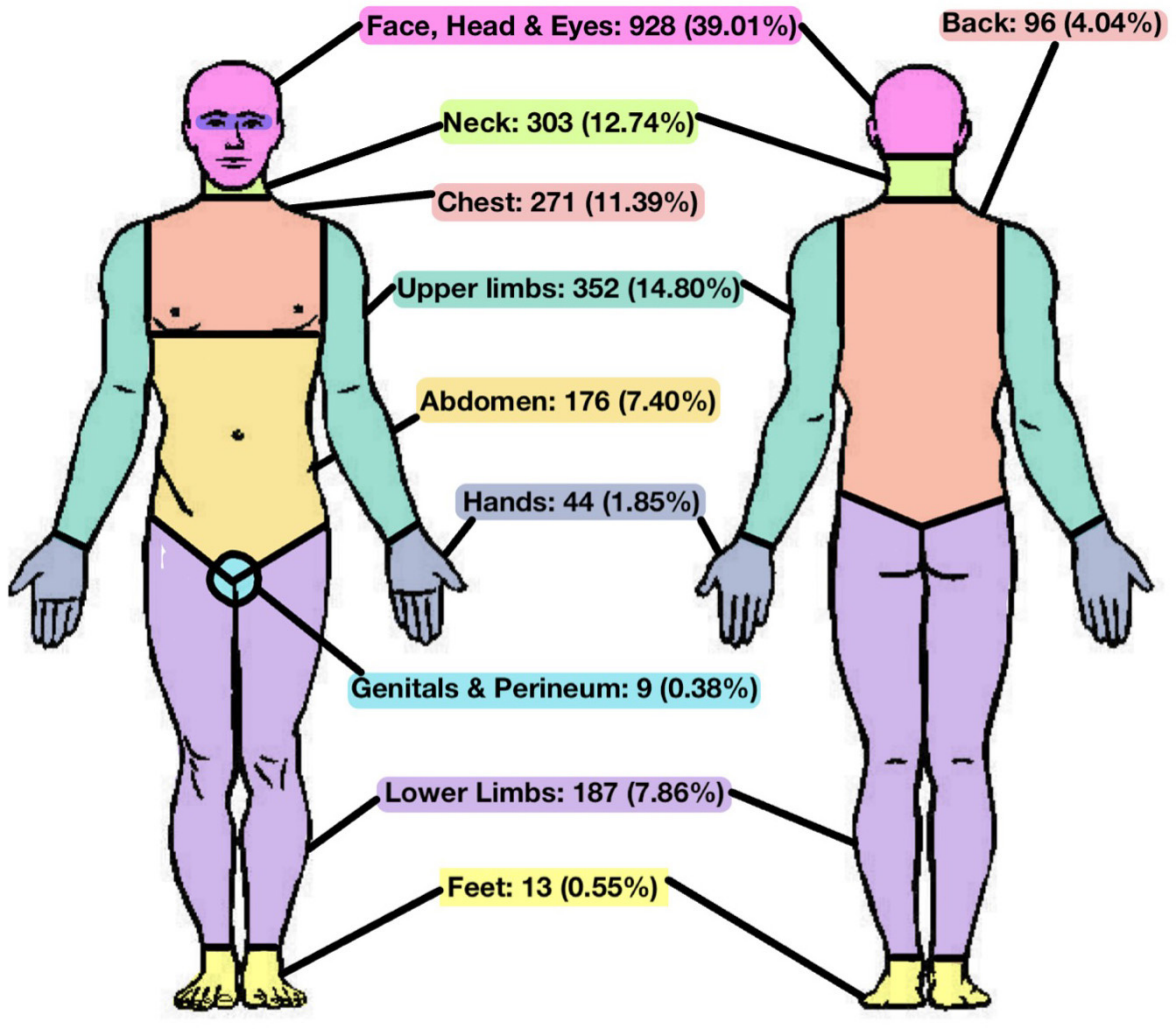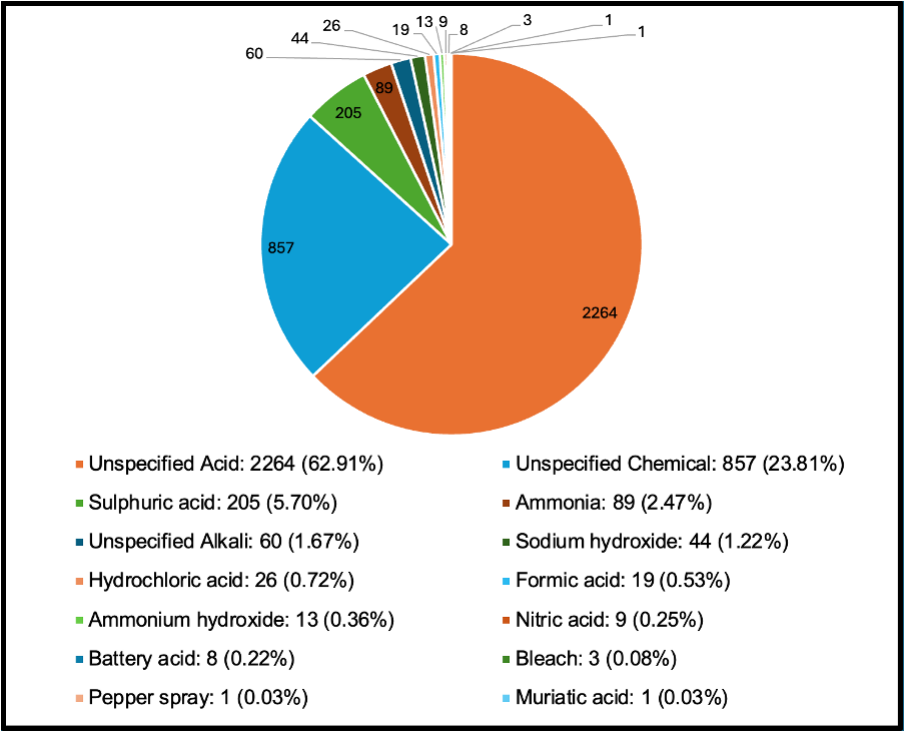# 897 A Global Analysis of Acid and Alkali Chemical Assault Burns (Vitriolage)

**DOI:** 10.1093/jbcr/iraf019.428

**Published:** 2025-04-01

**Authors:** John Warner-Levy, Zeeshan Sheikh, Karl Walsh

**Affiliations:** The University of Manchester; Manchester University NHS Foundation Trust; Manchester University NHS Foundation Trust

## Abstract

**Introduction:**

Chemical assaults involve the intentional use of chemicals to cause harm or injury, often with the aim of permanently disfiguring the victim. These attacks commonly occur in domestic settings, with reliable epidemiological data on this form of violence remaining scarce. This review seeks to explore key epidemiological aspects of chemical assaults, including their incidence across different regions, demographic trends, motives, and the types of substances used.

**Methods:**

Our review included all peer-reviewed reports that provided original cases of chemical assaults, detailing at least the number of victims involved.

**Results:**

Globally, the mean age of victims was 29.03 years (SD 11.99), and the mean TBSA was 14.12% (SD 15.42). Females were slightly more likely to be victims (50.76%), while males were more frequently the perpetrators (89.01%). Over half of the victims with a recorded education level were illiterate (53.76%), and homemakers were the most commonly affected occupation (33.84%). Chemical assaults most often occurred on the street (47.40%), followed by at home (42.81%), and were more likely to take place during the day rather than at night. Figure One displays the different substances used in assaults, of which acids were the most commonly used, accounting for 70.36% of cases. Victims were more likely to have a pre-existing relationship with their perpetrator (58.56%). Among these, romantic relationships accounted for 33.1%. The most frequent motive for chemical assaults was the denial of sex or love (13.82%), followed by land disputes (12.54%). As shown in Figure Two, the body parts most commonly burned were the head, face, and eyes (39.01%).

**Conclusions:**

Further research, particularly inter-country analysis could be used to develop more effective strategies to prevent chemical assaults and reduce their incidence.

**Applicability of Research to Practice:**

This research highlights key demographic patterns and motives behind chemical assaults, which can inform targeted prevention strategies such as stricter regulation of acid sales, public awareness campaigns on domestic violence, and community interventions to address underlying disputes, particularly in high-risk areas.

**Funding for the Study:**

This research received no specific grant from funding agencies in the public, commercial, or not-for-profit sectors.